# Microfluidic Platforms Designed for Morphological and Photosynthetic Investigations of *Chlamydomonas reinhardtii* on a Single-Cell Level

**DOI:** 10.3390/cells11020285

**Published:** 2022-01-14

**Authors:** Eszter Széles, Krisztina Nagy, Ágnes Ábrahám, Sándor Kovács, Anna Podmaniczki, Valéria Nagy, László Kovács, Péter Galajda, Szilvia Z. Tóth

**Affiliations:** 1 Institute of Plant Biology, Biological Research Centre, H-6726 Szeged, Hungary; szeles.eszter@brc.hu (E.S.); skovacs2@uni-koeln.de (S.K.); panna@cs-sld.com (A.P.); nagy.valeria@brc.hu (V.N.); kovacs.laszlo@brc.hu (L.K.); 2 Doctoral School of Biology, University of Szeged, H-6722 Szeged, Hungary; 3 Institute of Biophysics, Biological Research Centre, H-6726 Szeged, Hungary; nagy.krisztina@brc.hu (K.N.); abraham.agnes@brc.hu (Á.Á.); galajda.peter@brc.hu (P.G.); 4 Doctoral School of Multidisciplinary Medical Sciences, University of Szeged, H-6720 Szeged, Hungary

**Keywords:** cell cycle, *Chlamydomonas reinhardtii*, chlorophyll *a* fluorescence, microfluidics, non-photochemical quenching, photosynthesis, single-cell

## Abstract

*Chlamydomonas reinhardtii* is a model organism of increasing biotechnological importance, yet, the evaluation of its life cycle processes and photosynthesis on a single-cell level is largely unresolved. To facilitate the study of the relationship between morphology and photochemistry, we established microfluidics in combination with chlorophyll *a* fluorescence induction measurements. We developed two types of microfluidic platforms for single-cell investigations: (i) The traps of the “Tulip” device are suitable for capturing and immobilizing single cells, enabling the assessment of their photosynthesis for several hours without binding to a solid support surface. Using this “Tulip” platform, we performed high-quality non-photochemical quenching measurements and confirmed our earlier results on bulk cultures that non-photochemical quenching is higher in ascorbate-deficient mutants (*Crvtc2-1*) than in the wild-type. (ii) The traps of the “Pot” device were designed for capturing single cells and allowing the growth of the daughter cells within the traps. Using our most performant “Pot” device, we could demonstrate that the F_V_/F_M_ parameter, an indicator of photosynthetic efficiency, varies considerably during the cell cycle. Our microfluidic devices, therefore, represent versatile platforms for the simultaneous morphological and photosynthetic investigations of *C. reinhardtii* on a single-cell level.

## 1. Introduction

Green algae are of outstanding ecological and increasing biotechnological importance for the production of high-value compounds, biostimulants, chemicals, biofuels, and so on. *Chlamydomonas reinhardtii* has become an excellent model system for green algae. It is a haploid and it is the only photosynthetic organism that is suitable for the transformation of its nuclear, chloroplast, and mitochondrial genome. Recent developments in genetic tools make it also an ideal model organism [1,2]. *C. reinhardtii* is also very often used to study photosynthesis and biogenesis of the chloroplast, mostly due to its capability for heterotrophic growth, thus even mutants severely affected in photosynthesis are viable [3].

Traditionally, experiments on algae are carried out on bulk cultures in which individual cells are continuously subjected to varying light intensities, oxygen concentrations, and nutrient supply as the culture age. Part of these problems can be solved by synchronizing the algal cultures and placing them in continuous cultivation systems [4]. However, in many cases, for instance, for developmental biology research, it would be preferential to study the phenotypes of single cells for several hours or throughout their lifetimes under identical physiological conditions. For this, microfluidics offers an ideal solution.

Fluid flow in microfluidic devices is typically characterized by low Reynolds numbers, which implies the formation of even and laminar flow fields in these devices. Therefore, chaotic changes in pressure and flow velocity characteristic to turbulent flows are lacking. This makes the precise engineering of the flow field possible through the geometry of the device and the pressures applied to drive the flow. In a carefully designed and applied microfluidic device, shear stress on cells can be minimized, enabling physiological experiments [5,6]. Furthermore, with such low Reynolds number flows, inertial forces may be neglected. With the advent of microfluidics, it also became possible to reduce laboratory-sized equipment to the scale of a microscope slide allowing massive parallelization of experiments with extremely low sample volumes and enabling experiments on a single-cell level [7].

In this study, we designed, constructed, and employed two types of microfluidic devices for single-cell analysis of *C. reinhardtii*. In addition to morphological examinations by microscopy, chlorophyll (Chl) *a* fluorescence induction measurements were also conducted, thus a complex physiological assessment of *C. reinhardtii* cells trapped in microfluidic devices became possible. The traps of the “Tulip” device are suitable for capturing and immobilizing individual cells enabling the measurement of their photosynthetic activity for several hours without binding to a solid support surface. The traps of the “Pot” devices capture single cells and enable cell divisions while retaining the daughter cells.

## 2. Materials and Methods

### 2.1. Design and Construction of Microfluidic Devices for C. reinhardtii

The microfluidic devices were constructed using standard photolithography and soft lithography techniques [8]. The devices were designed in KLayout, an open-source software (www.klayout.de, accessed on 17 November 2022). To fabricate the master molds, for each device a silicon wafer was coated with thin films of the negative photoresist SU-8 (Kayaku Advanced Materials, Inc., Westborough, MA, USA) at a defined thickness, and the design of the device was exposed in the resin with a laser pattern generator (µPG 101, Heidelberg Instruments Mikrotechnik GmbH, Heidelberg, Germany).

The master mold for the “Tulip” microfluidic device was fabricated from a single SU-8 layer (SU-8 2005 resin, Kayaku Advanced Materials, Inc., Westborough, MA, USA) with a thickness of 6.8 (SD = 0.8) µm. The SU-8 molds were silanized overnight under vacuum using tridecafluoro-1,1,2,2-tetrahydrooctyl-trichlorosilane (Gelest Inc., Morrisville, PA, USA). The “Pot” devices were fabricated by applying two SU-8 layers with different thicknesses and writing two different patterns into them one after the other [9]. This two-layered structure is important to reach sufficient depth for the chambers to perform long-term experiments on dividing cells but also for having shallow and narrow gaps that prevent cells from escaping the traps. The first layer of the “Pot” traps is 3.8 (SD = 0.5) µm thick (fabricated from SU-8 2005 resin) and it contains the narrow notches (<3 µm). The second layer is about 7.5 (SD = 0.6) µm thick (made of SU-8 2007 resin) and contains the continuous wall of the traps without any gaps.

Polydimethylsiloxane (ratio 10:1 PDMS: curing agent; Sylgard 184, DowInc., Midland, MI, USA) was poured over the SU-8 molds and left to cure overnight at 40 °C. Access holes for fluid inlets/outlets tubing were punched into the cured and cut PDMS pieces. The PDMS piece is then closed off by irreversibly binding it to a microscope slide by oxygen plasma treatment. Finally, we treat our microfluidic devices at 85 °C for 30 min. Liquid cell cultures, buffers, etc. are introduced into the microfluidic chips via tubings plugged into the inlet holes and fluid flow was driven and controlled by syringe pumps (Model No. 4000, New Era Pump Systems Inc., Farmingdale, NY, USA).

### 2.2. Characterizing the Properties of the Fluid Flow by Model Calculations and Tracer Particles

The characteristic properties of the fluid flow within the microfluidic devices were calculated with Comsol Multiphysics 4.3a software (COMSOL AB, Stockholm, Sweden). The velocity magnitude profiles were calculated by the “Laminar flow” model using a time-dependent study. For the “Tulip” device we applied a “shallow channel” approximation to get a quasi 3D model of the streamlines. In the case of the “Pot” device (Type II and VI), we built a 3D model regarding its two-layered geometry (Supplementary Figure S2). The “Tulip” and the “Pot” devices consist of 3 and 7 parallel channels, respectively, that we took into account when building the flow model. Therefore, we used 26.7 µL/h (“Tulips”) and 17 µL/h (“Pot”) flow rates in the calculations.

Besides the model calculations, we used fluorescent microspheres (Fluoresbrite YG carboxylate microspheres, d = 1 µm; Polysciences, Inc. Warrington, PA, USA) as tracer particles to visualize the streamlines of the flow, which gave us the same result as the Comsol model.

### 2.3. Chlamydomonas reinhardtii Strains and Cell Culture Conditions

To test the “Tulip” microfluidic device, an ascorbate-deficient mutant (*Crvt2-1*), originating from the CLiP collection and characterized in detail [10] was used, along with its appropriate wild-type strain, CC-4533. Precultures of CC-4533 and *Crvt2-1* mutant strains were grown mixotrophically in Tris-acetate-phosphate (TAP) medium in 25-mL Erlenmeyer flasks for three days on a rotatory shaker at 22 °C and 80 µmole photons m^−2^s^−1^. The precultures were transferred to TAP or high salt minimal (HSM) media. The initial Chl content of the main culture was set at 0.1 µg Chl(a + b)/mL. By the third or fourth day of growth in TAP or HSM media, respectively, they reached a cell density of 2–4 million cells/mL.

For testing the “Pot” device, the widely used CC-124 wild-type strain was employed. Precultures were grown mixotrophically in TAP medium in 50-mL Erlenmeyer flasks for three days on a rotatory shaker at 22 °C and 80 µmole photons m^−2^s^−1^. The precultures were diluted to 0.1 µg Chl(a + b)/mL and were partially synchronized in a Multi-Cultivator MC 1000-OD instrument (Photon Systems Instruments, Brno, Czech Republic) at 22 °C, 200 µmole photons m^−2^s^−1^, bubbled with air containing 1% CO_2_ with 18 h light/6 h dark cycles. By the second day of growth in TAP media the cultures reached a Chl content of 4–7 µg Chl(a + b)/mL, corresponding to 2–4 million cells/mL.

### 2.4. Cell Loading and Culturing in the Microfluidic Devices

Two separate units of the “Tulip” microfluidic device were loaded with CC-4533 and *Crvtc2-1* cultures of 1 µg Chl(a + b)/mL in TAP or HSM medium, at a flow rate of 80 µL/h, provided by a syringe pump (Model No. 4000, New Era Pump Systems Inc.,Farmingdale, NY, USA). The flow rate was kept constant during the entire experiment.

For filling up the “Pot” microfluidics device containing seven different trap types, we loaded the CC-124 algae culture (1 µg Chl(a + b)/mL) at a flow rate of 80 µL/h for 60 min using a syringe pump. Following this step, TAP medium was provided continuously, at a flow rate of 180 µL/h until the end of the experiment. For filling up the “Pot” microfluidics device with Traps II and VI, we set an initial flow rate of 80 µL/h for approx. 40 min and then the inlet tube was flushed with TAP medium and then the flow rate was kept at 180 µL/h until the end of the experiment. 

Illumination was provided by white LED spot microscope lamps at an intensity of approx. 180 µmole photons m^−2^s^−1^ on the surface of the microfluidic device with 18 h light/6 h dark cycles. In order to prevent bacterial contamination, Hygromycin B (Duchefa Biochemie, Haarlem, The Netherlands) and paromomycin sulfate (Sigma Aldrich, Burlington, MA, USA) were added to the TAP medium at final concentrations of 1 µg/mL each before loading the cells into the microfluidic device.

### 2.5. Microscopy and Chl a Fluorescence Measurements

Bright-field images were captured by an Axiocam 503 color CCD camera mounted to the microscope with a 60N-C 2/3” 0.63× video adapter (Zeiss GmbH, Jena, Germany). We used the Microscopy version of the Imaging PAM M-series Chl *a* fluorometer coupled to an AxioScope A1 microscope (Zeiss GmbH) to measure various photosynthetic parameters. For visualization, 20× (Zeiss, Fluar 20×/0.75) and 63× objectives were used (Zeiss, Plan-Neofluar 63×/1.25 Oil). 

Cells were dark-adapted for 15 min prior to fluorescence measurements. Chl *a* fluorescence was induced by a modulated blue (470 nm) measuring light and the emitted fluorescence image was captured by an IMAG-K6 CCD camera (Walz GmbH, Effeltrich, Germany) mounted to the microscope via a 60N-C 2/3” 0.5× video adapter. For the determination of the F_0_ level of Chl *a* fluorescence, the measuring light intensity was set at a value of 2 and frequency to 2 Hz. The gain was set to step 20 and damping was set to step 1 and 5 for 20× and 63× objectives, respectively. F_M_ values were obtained by an 820 ms saturating blue light pulse at an intensity value of 5. F_V_/F_M_, an indicator of photosynthetic efficiency was calculated as (F_M_ − F_0_)/F_M_. For NPQ induction, blue actinic light of about 151 and 383 µmole photons m^−2^s^−1^ was applied for 30 min and F_M_’ was obtained upon saturation pulses provided every min. NPQ was calculated as (F_M_ − F_M_’)/F_M_’. On the cessation of actinic blue light, cells were exposed to a continuous far-red illumination obtained from an external LED panel and the recovery of fluorescence was monitored for 30 min.

F_V_/F_M_ values on batch cultures were determined using a Handy-PEA instrument (Hansatech Instruments Ltd., King’s Lynn, UK). *Chlamydomonas reinhardtii* cultures were dark-adapted for about 15 min, and then 60 µL of cell suspension (150 µg Chl(a + b)/mL) was placed onto a Whatman glass microfibre filter (GF/B) that was placed in a Handy-PEA clip and measured. The light intensity was set at 3500 µmole photons m^−2^s^−1^ and the duration of the measurement was 1 s.

### 2.6. Electron Microscopy

The depths and other characteristic parameters of the microfluidic devices were checked by scanning electron microscopy (SEM). For this purpose, PDMS pieces were coated by thin a layer of gold by a Quorum Q150T sputter coater (100 mA, 120 s) and then placed into and examined with a JSM-7100F field emission scanning electron microscope (using 5 kV voltage).

### 2.7. Statistical Analysis

All experiment was repeated four to eleven times, and averages are shown with standard errors or representative examples are shown, when appropriate. Student *t*-test, mixed designed two-way ANOVA, and one-way ANOVA with Dunnett multiple comparison tests were performed.

## 3. Results

For the construction of microfluidic devices, we used soft lithography, which is a widespread technique in microfluidics [8,9]. First, a master mold was fabricated by photolithography, then replica moldings were produced in polydimethylsiloxane (PDMS). PDMS is a silicon-based flexible organic polymer. It is biologically inert, optically clear, and chemically resistant. It is also gas permeable, therefore, it can ensure sufficient oxygen or carbon dioxide diffusion to the cells. PDMS is transparent for UV, visible, and near-infrared light, making it suitable for various microscopy imaging techniques. Due to its relatively low background fluorescence, it also enables fluorescence microscopy applications [11,12], including steady-state Chl *a* fluorescence imaging [13,14,15]. To facilitate the study of the relationship between morphology and photochemistry in *C. reinhardtii*, we employed microfluidics in combination with Chl *a* fluorescence induction measurements.

### 3.1. “Tulip” Microfluidics Platform for Long-Term Chl a Fluorescence Measurements on Single Cells

**Figure 1:**
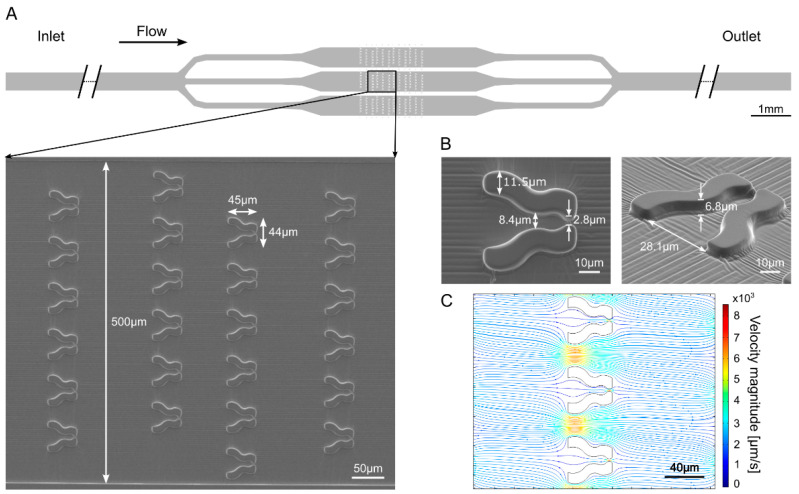
“Tulip” microfluidics platform for capturing and immobilizing individual *C. reinhardtii* cells enabling the measurement of their photosynthetic activity. (**A**) Scheme of the device and a scanning electron microscopy image taken in a region of the schematic view. The direction of the flow is indicated by the arrow. (**B**) Scanning electron microscopy images of single “Tulip” traps in the microfluidic device. (**C**) Computational modeling of the flow in the device. The density of the streamlines and the color code represent the velocity magnitude.

The aim of constructing the “Tulip” microfluidic device was to trap and immobilize individual cells for a few hours enabling high-quality Chl *a* fluorescence measurements. The device consists of three parallel channels in which the traps are organized in arrays (Figure 1A). The traps have a relatively wide opening (of about 28 µm), a narrower middle section (about 8.5 µm in diameter), and a narrow exit for the outflow of culture media (about 3 µm) that prevents cells from escaping (Figure 1B). The height of the traps is about 7 µm to limit cell movement in the vertical direction (Figure 1B) for the Chl *a* fluorescence measurements. These traps are arranged in multiple rows, and the subsequent rows are laterally shifted with respect to each other. An array of 72 traps is placed within a 500 μm wide channel and three parallel channels accommodate a total of 216 traps (Figure 1A). Two such units were constructed within the same microfluidic device, enabling the investigation of two algal strains in parallel.

Computational modeling of the flow field demonstrates that in the case of empty traps the magnitude of the flow velocity is about 10-fold lower at the entrance of the trap compared to the velocity between neighboring traps (Figure 1C, Supplementary Figure S1). Due to the fact that the inter-trap separation distance and the width of the trap entrance are similar, about 10% of the flow goes through the traps, enabling filling up the traps with cells. Trapping efficacy is further increased by the lateral shift of subsequent rows as the inter-trap flow hits a trap in the next row with about 10% probability (Figure 1C, Supplementary Figure S1).

**Figure 2:**
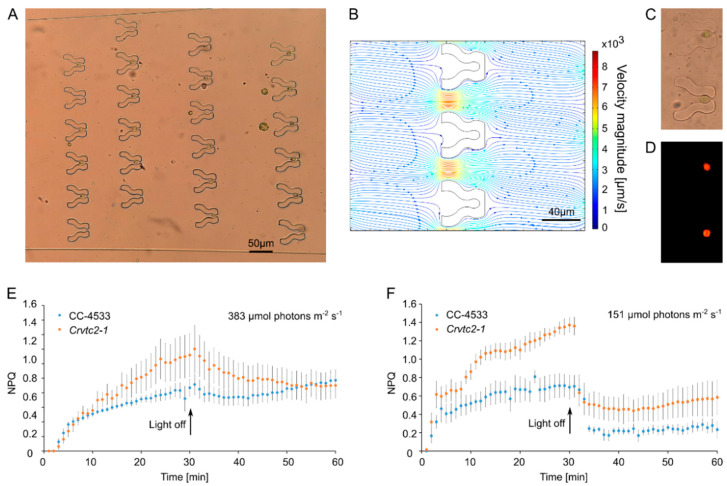
Capturing *C. reinhardtii* cells and non-photochemical quenching (NPQ) measurements in the “Tulip” microfluidics platform. (**A**) About 70% of the traps are occupied by single cells in the device. Images were taken using a Fluar 20×/0.75 objective. (**B**) Computational modeling of the flow when the outlet is blocked by a cell. The density of the streamlines and the color code indicate the velocity magnitude. (**C**) Bright-field microscopy image of the trapped cells. Images were taken using a Plan-Neofluar 63×/1.25 Oil objective. (**D**) Maximum Chl *a* fluorescence (F_M_) measurement of the captured cells in the “Tulip” device, taken by the Microscopy version of Imaging PAM and using a Plan-Neofluar 63×/1.25 Oil objective. (**E**) NPQ measurements on individual wild-type (CC-4533) and ascorbate-deficient *Crvtc2-1* cells, grown and measured in TAP medium at about 383 µmol photon m^−2^s^−1^. (**F**) NPQ measurements on individual wild-type (CC-4533) and ascorbate-deficient *Crvtc2-1* cells, grown and measured in HSM medium at about 151 µmol photons m^−2^s^−1^. The results represent an average of five to seven measurements with their standard errors.

Figure 2A demonstrates that upon flowing a cell culture through the device a large majority (about 65–71%) of traps captured a single cell; the proportion of traps capturing multiple cells was negligible. This is the consequence of the fact that a cell within the trap effectively blocks the flow through the trap which dramatically reduces the chance of entrance for another cell (Figure 2B). The traps embraced the cells in their middle sections and acted as a physical immobilizing constraint facilitating Chl *a* fluorescence measurements (Figure 2C,D). 

Next, non-photochemical quenching (NPQ) measurements were carried out on an ascorbate-deficient mutant, called *Crvtc2-1*, with about 10% ascorbate relative to the wild-type (CC-4583; [10]. Non-photochemical quenching was first determined in mixotrophically grown cultures (in TAP medium), and at relatively strong blue light (about 383 µmole photons m^−2^s^−1^). We found that NPQ was higher in the ascorbate-deficient mutant than in the CC-4583 wild-type strain, and NPQ relaxation was very slow in both strains in weak far-red light—these observations are in broad agreement with our earlier results obtained on bulk cultures, in red light (Figure 2E; [10]). NPQ of the *Crvtc2-1* mutant was about twice as high as in the wild-type when grown under photoautotrophic conditions in HSM medium and assessed at about 151 µmole photons m^−2^s^−1^ blue light (Figure 2F, Table 1). NPQ relaxation of the photoautotrophic cultures was rapid, in agreement with [10]. From a technical point of view, we emphasize that NPQ values were stable throughout the 60 min-measurements. 

We also determined the F_V_/F_M_ values on batch cultures using the fast Chl *a* fluorescence (OJIP) technique and on single cells in our microfluidics platform, originating from the same algae culture. We found no significant differences between the F_V_/F_M_ values of the two strains grown in TAP or HSM media (Table 1). Thus, the “Tulip” microfluidics platform enabled the reliable assessment of NPQ without binding the cells to a solid support surface that is normally a necessity when imaging Chl *a* fluorescence, with the risk of damaging or perturbing the cells. Due to the possibility of up-scaling (i.e., creating more channels and traps on a microfluidic chip) this type of microfluidic trapping would be particularly useful to study photosynthetic heterogeneity within a cell population.

### 3.2. “Pot” Microfluidics Platform for Observing Cell Division

Observation of the growth and division of the mother cell and the development of its progeny are usually performed on synchronized cultures in which the majority of cells are in the same phase of the cell cycle (e.g., [16,17]). Although this type of research has provided valuable insights into the details of the cell cycle, precise control of culture parameters and investigating the division of unique cells could enlighten further details of cell division. Combining morphological assessment with Chl *a* fluorescence induction measurements could, for instance, reveal how photosynthetic activity changes during the cell cycle.

When designing microfluidic devices for studying cell division, a major challenge is to capture and retain the mother cells and the daughter cells within the same traps. The typical diameter of a mature *C. reinhardtii* cell is in the range of 8 to 10 µm (e.g., [18]), and the daughter cells are much smaller (e.g., [19]).

**Figure 3:**
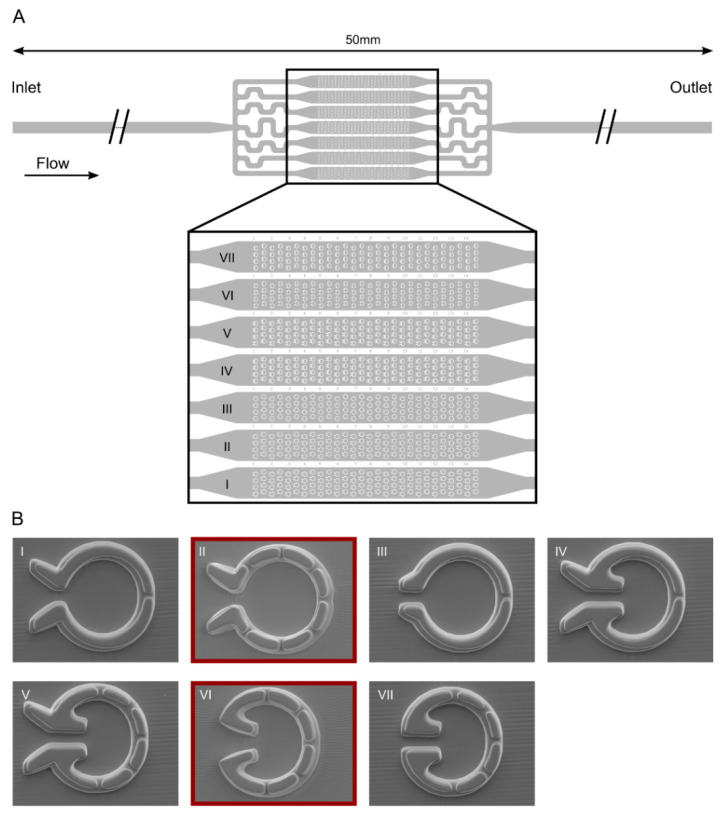
“Pot” microfluidics platform for capturing individual *C. reinhardtii* cells enabling cell division and the measurement of their photosynthetic activity. (**A**) Scheme of the device with seven different types of traps located in parallel channels. (**B**) Scanning electron microscopy images of the individual traps.

We designed seven types of so-called “Pot” traps, arranged in arrays within parallel channels in a microfluidic device (Figure 3). These traps differed in the inlet geometry (both on the inner and outer side of the trap entrance) and the number of outlet slits. We constructed the device of two layers with 4 and 7.5 µm thickness, respectively. This way, it was possible to keep the width and depth of the slits low enough (about 3 and 4 µm, respectively) to retain the smaller daughter cells while ensuring a larger depth (12 µm) of the trap itself to accommodate the mother cells without much physical constraint.

**Figure 4:**
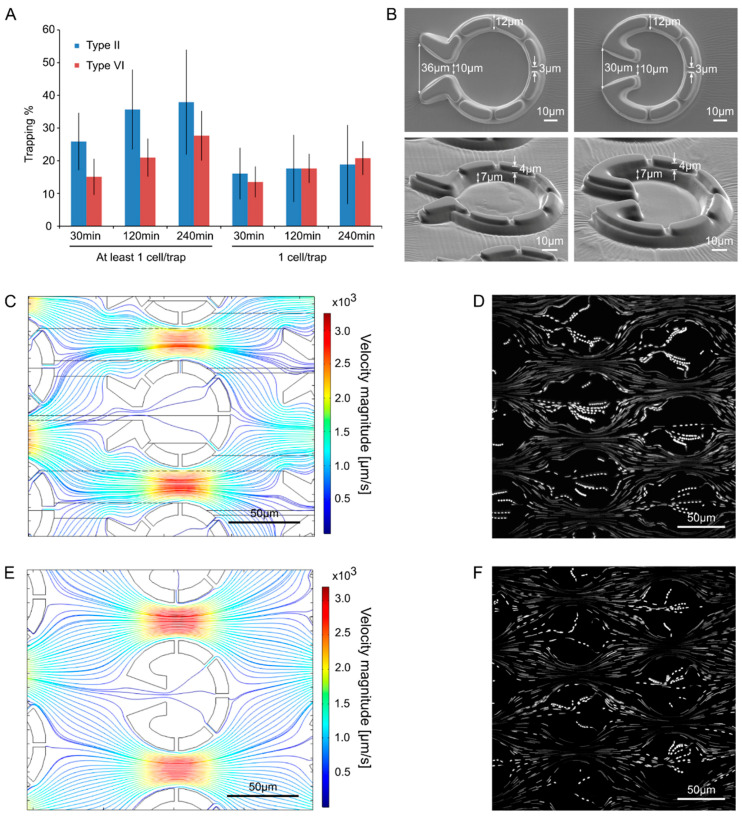
Comparison of “Pot” trap Types II and VI. (**A**) Comparison of the trapping efficiency. The cell loading lasted for 60 min and the trapping efficiency was assessed at 30, 120, and 240 min. The results represent the averages of three independent experiments with their standard error. No significant differences were detected between Types II and VI (Student *t*-test *p* < 0.05). (**B**) Scanning electron microscopy images of the traps from above and at tilted angles. (**C**) Computational modeling of the flow in trap Type II. The density of the streamlines and the color code represent the velocity magnitude. (**D**) Streamlines of the fluid flow visualized by fluorescent microbeads (1 µm) in trap Type II. (**E**) Computational modeling of the flow in trap Type VI. The density of the streamlines and the color code represent the velocity magnitude. (**F**) Streamlines of the fluid flow visualized by fluorescent microbeads (1 µm) in trap Type VI.

The traps were evaluated based on the percentage of traps capturing single cells. Preliminary experiments demonstrated that the most efficient geometries were Types II and VI; the least efficient one was Type I, and the rest showed intermediate characteristics. For the Type II geometry, the percentage of traps capturing at least one cell/trap was about 25% after 30 min of loading, and this value increased to about 35% after 240 min of loading (Figure 4A). For type VI, the percentage of traps capturing at least one cell was slightly lower (about 15% and 28% after 30 min and 240 min of loading, respectively). However, the proportion of cells capturing exactly one cell was equal in Types II and VI (15 to 20%, Figure 4A).

Both trap types possess a relatively wide opening (36 and 30 µm) with a funnel shape, and they have several outlet slits that are about 3 µm wide and about 4 µm deep (Figure 4B). Furthermore, they both have concave geometries on the inner side of the entrance to divert swimming cells within the trap away from the inlet. The observation that the percentage of traps capturing ≥1 cell was slightly higher in Type II, could be explained by its wider opening, as well as by the extra two outlet slits (seven vs. five slits in Type II and Type VI geometries). The wider opening may increase the chance of a cell moving into the entrance funnel. At the same time, the extra slits slightly increase the flow through the trap which may also increase trapping efficiency. Computational modeling of the flow fields (Figure 4C,E, Supplementary Figure S3) and measurements using tracer particles (microbeads with a diameter of 1 µm, Figure 4D,F) show that for both traps the medium flow is very fast in between the traps, whereas at the opening and especially within the traps, it is much slower. This is due to the flow resistance of the small slits of the traps.

**Figure 5:**
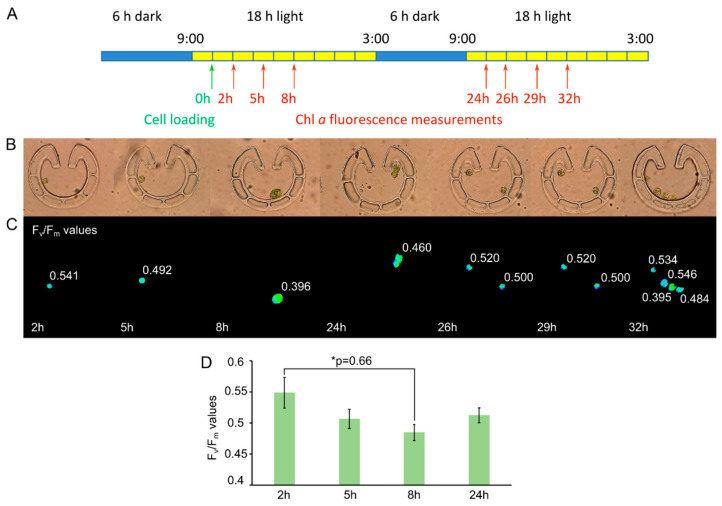
Cell division in “Pot” trap Type VI. (**A**) Scheme of the dark–light cycles and the time of cell loading (green arrow) and Chl *a* fluorescence measurements (red arrows). (**B**) Bright-field images of the traps with *C. reinhardtii* cells at the indicated times. (**C**) F_V_/F_M_ values taken at the indicated times. A representative example is shown. Images were taken by using a Plan-Neofluar 63×/1.25 Oil objective. (**D**) Averages of F_V_/F_M_ values (n = 10 to 13) as determined in (**C**), originating from six independent experiments. One-way ANOVA with Dunnett multiple comparison test using the 2-h sample as control indicates significant differences at *p* < 0.1 level.

Since the probability that only one cell is captured per trap (and not multiple cells, Figure 4A) is mildly higher in the case of Type VI, we decided to use this type of “Pot” device in the follow-up experiments. Cell cycle was monitored in cells originating from synchronized cultures grown in light–dark cycles, for 48 h. After loading the device in the morning (Figure 5A), cells started to grow in size. In parallel, their F_V_/F_M_ value decreased by the end of the 8th h (to about 0.4, a representative example is shown in Figure 5C and the averages are in Figure 5D). Cell division occurred by the 24th h after loading, and the daughter cells remained inside the traps—their F_V_/F_M_ values were relatively high (about 0.51). These data demonstrate that the cell cycle of *C. reinhardtii* is accompanied by changes in photosynthetic efficiency.

## 4. Discussion

Most work to study algal physiology has been performed on bulk liquid cultures, which typically contain a few million cells per milliliter. The obtained macroscopic parameters average a heterogeneous cell population, in which the cells may be in various phases of their cell cycle and/or experience different environmental conditions. This averaging may obscure features that may be essential for the understanding of the investigated physiological processes. In contrast to these conventional cultivation methods and measuring tools, microfluidic cultivation systems represent an excellent alternative to study individual cells or a small number of cells in a well-defined environment, in situ and in real-time. Another major feature of microfluidic technology is the precise and dynamic control of the cellular microenvironment; this is a remarkable advantage compared to bulk cultures, in which media exchange requires centrifugation, representing stress effects for the algal cells. In comparison with flow cytometry, the main advantage of microfluidics is that it enables examining essential features of algae, such as cell division rate and various physiological responses [20]. Furthermore, flow cytometry sorting is accompanied by large hydrodynamic stress, resulting in a low recovery rate after sorting [20].

Several microfluidic devices have been developed for algae (for a review, see [21]) including platforms for sorting of algal cells with different shapes, sizes, improved phototaxis, for performing systematic growth studies, medium screening, and for separating bacteria from algae [22,23,24,25,26,27,28]. A complex microfluidic platform for trapping and releasing single *C. reinhardtii* cells was also constructed [29].

Monitoring steady-state Chl *a* fluorescence of algal cells has been proven particularly useful for algae-based pesticide detection [30,31] and determining the level of pollutions [32]. Microfluidic devices in combination with steady-state Chl *a* fluorescence were also employed to measure and compare the growth dynamics and biomass differences between various species and to study the cellular development of microalgae [33,34,35].

Recently, Behrendt et al. [36] developed “PhenoChip”, a compact and versatile microfluidic platform to enable rapid, high-throughput phenotyping of *Symbiodinium* and cyanobacteria based on their photosynthetic performance under relevant environmental conditions. By applying the Microscopy version of the Imaging PAM M-series Chl *a* fluorometer, they could determine the F_V_/F_M_ values of single *Symbiodinium* cells, and use it to identify cells with elevated resilience toward high temperature.

Microfluidics devices available for *C. reinhardtii* enabled only steady-state fluorescence measurements so far. Devices for *C. reinhardtii* are available almost exclusively for studying a micropopulation of cells (e.g., [37]) and rarely single-cell analysis [38]. To fill this gap, we constructed two types of microfluidic devices specifically for *C. reinhardtii*, enabling a high-quality assessment of photosynthesis on a single-cell level. Our devices are relatively easy to fabricate and assemble, they can be operated by simple flow control techniques and do not require pressurization control of pneumatic components.

In the case of microfluidic cell traps operating with fluid flow, there are two requirements. In the empty state of the trap, a substantial portion of the liquid flow should travel through the trap. This traverse flow can carry cells into the trap from a flowing culture. However, flow through an occupied trap should be small, to minimize the chance of multi-cell trapping. In the case of the “Tulip” device, the culture flows through a wide opening and leaves through a narrow slit that forms the bottleneck for the flow. While the dimensions of the slit determine the effective flow rate through the “Tulip” trap, the width and depth need to be set by taking into account the average *C. reinhardtii* cell size. The traps of the “Tulip” device proved to be very efficient in catching single cells from a flowing culture. The wide entry section acts as a funnel, guiding cells towards the inner section of the trap. Trapped cells effectively block the flow through the trap itself thereby preventing the accumulation of further cells in the trap. These locked-in cells are embraced tightly by the curved walls and ensure a very stable position with negligible translational and rotational movement. The “Tulip” traps also restrict vertical movements of the cells thereby high quality and reproducibility of Chl *a* fluorescence measurement is ensured. Thus, immobilization of individual cells is achieved without the necessity to remove the flagella or glue the cells to a solid surface that could result in severe stress effects. 

We obtained high-quality NPQ kinetics on single *C. reinhardtii* cells (Figure 2). In the future, the “Tulip” platform could be particularly useful to study (1) population heterogeneity upon various treatments and (2) the effects of inhibitors, pollutants, and other compounds on the morphology and photosynthesis of *C. reinhardtii*. Evidently, not only NPQ but any other parameter available in the Imaging PAM M-series Chl *a* fluorometer software could be determined, on the timescale of seconds to several hours. 

The “Pot” platform was designed for a different purpose, that is to catch single cells and retain their progeny for prolonged times (for the duration of one or two cell cycles). Cells in different phases of the cell cycle have an effective diameter of 3 to10 µm. Cells are immotile during division but can swim with up to 40 µm/s in other phases of the cell cycle [39]. To accommodate several progeny cells, these traps have the shape of a small chamber, or “pot”, rather than a tight, single cell-sized cavity. However, similarly to the “Tulip” traps, a wide opening and outlet slits facilitate a through-flow that is necessary for the initial loading of cells into the traps. Upon testing different geometries, a funnel-shaped entry facing the incoming flow and several small slits on the transversal side proved to be the most effective. Small curved wall segments are applied next to the inner sides of the entrance to guide the cells away from the entrance and reduce the chance of cells swimming out of the trap. As in the case of the “Tulip” traps, the slits are the main contributors to the flow resistance across the traps. More slits facilitate a higher flow rate through the traps increasing the probability of cell trapping during the initial filling process. In addition, a sustained through flow counteracts the swimming motility of cells, reducing the chance of cells swimming out of the trap. In order to keep the small daughter cells inside the traps, the size of the outlet gaps was minimized by constructing the device of two layers. The height of the traps is large enough (about 11 µm) so that cells can move around and cell divisions take place. The assessment of the basic F_0_, F_M_ and F_V_/F_M_ parameters (and their counterparts in the light-adapted state) is possible, but no long-term fluorescence kinetics measurements can be taken in the “Pot” device. 

Using the “Pot” microfluidics platform, we demonstrated that photosynthetic efficiency changes during the cell cycle, which is in line with earlier photosynthetic activity measurements [40] and transcriptomics data [41,42]. Stenkert et al., [42] observed that Chl biosynthetic genes peak during the first half of the day that precedes the observed rise in cellular Chl content. Most genes involved in photosynthetic electron transfer were also expressed during the day before cell division takes place in the dark. This raises the possibility that the photosynthetic apparatus undergoes some sort of remodeling upon cell and chloroplast division. We suggest that single-cell analysis in microfluidic devices is a powerful tool to study this hypothesis, in combination with employing phenotypic image analysis with computational tools (e.g., [43]). 

We note that the platforms presented here do not enable collecting individual cells after the analyses. However, based on our preliminary experiments and literature data, we suggest that the most straightforward way is to catch individual cells with laser tweezers [44] and to place them into tiny reservoirs within the microfluidic device, from which they could be retrieved later on for further analyses. 

## Figures and Tables

**Table 1:** F_V_/F_M_ and NPQ values of CC-4533 and *Crvtc2-1* cultures grown in HSM or TAP media. F_V_/F_M_ measurements were carried out on a batch culture, using a Handy-PEA fluorometer or on single cells in the “Tulip” microfluidic platform. NPQ values were obtained in the “Tulip“ microfluidic platform, after 30 min of light adaptation to 383 µmol photons m^−2^s^−1^ (TAP) or 151 µmol photon m^−2^s^−1^ (HSM).

		CC-4533		*Crvtc2-1*	
		TAP	HSM	TAP	HSM
F_V_/F_M_	Batch culture	0.467 ± 0.016 (n = 9)	0.429 ± 0.024 (n = 4)	0.500 ± 0.024 (n = 8)	0.426 ± 0.024 (n = 4)
	Single cell	0.473 ± 0.016 (n = 9)	0.448 ± 0.026 (n = 4)	0.481 ± 0.017 (n = 8)	0.469 ± 0.026 (n = 4)
NPQ		0.962 ± 0.105 (n = 9)	0.705 ± 0.119 (n = 4)	1.868 ± 0.392 * (n = 5)	1.360 ± 0.096 * (n = 4)

No significant differences of F_V_/F_M_ could be detected by mixed designed two-way ANOVA at *p* < 0.05 level. * indicates significant differences between the means of NPQ of the CC-4533 and *Crvtc2-1* cultures obtained in the same media by Student *t*-test (*p* < 0.05).

## Data Availability

All data presented in this study are available within this article or Supplementary Materials. There are no special databases associated with this manuscript.

## Supplementary Materials

The following are available online at https://www.mdpi.com/article/10.3390/cells11020285/s1, Figure S1: Calculated flow field in the “Tulip” microfluidic device. The density of the streamlines and the color code represent the velocity magnitude. (A,B) streamlines with empty traps (without trapped cells) and the corresponding velocity magnitude profile across the channel along the line. (C,D) streamlines with closed traps (simulating traps occupied by cells) and the corresponding velocity magnitude profile across the channel along the line. Figure S2: Schematic representation of the 3D computational model for the two-layered “Pot” microfluidic device. (A) Scheme of the two layers for Type VI. (B) The applied geometry in the Comsol simulations shows the layered structure and the direction of the fluid flow. Figure S3: Computational modeling of the fluid flow in the “Pot” microfluidic device. The density of the streamlines and the color code represent the velocity magnitude. (A,B) flow lines at 7.6 and 1.9 µm depth in case of Type VI traps and the corresponding velocity magnitude profile across the channel along the line; (C,D) flow lines at 7.6 and 1.9 µm depth in case of Type II traps and the corresponding velocity magnitude profile across the channel along the line.
