# Supplementary material for: Microfluidic Platforms Designed for Morphological and Photosynthetic Investigations of Chlamydomonas reinhardtii on a Single-Cell Level
Source: Cells. 2022 Jan 14;11(2):285. doi: 10.3390/cells11020285 (PMC8774182; doi:10.3390/cells11020285)
Supplement: Supplementary file 1 [file cells-11-00285-s001.zip › cells-1507181-supplementary/Supplementary/SupplFigS1_v03.pdf]

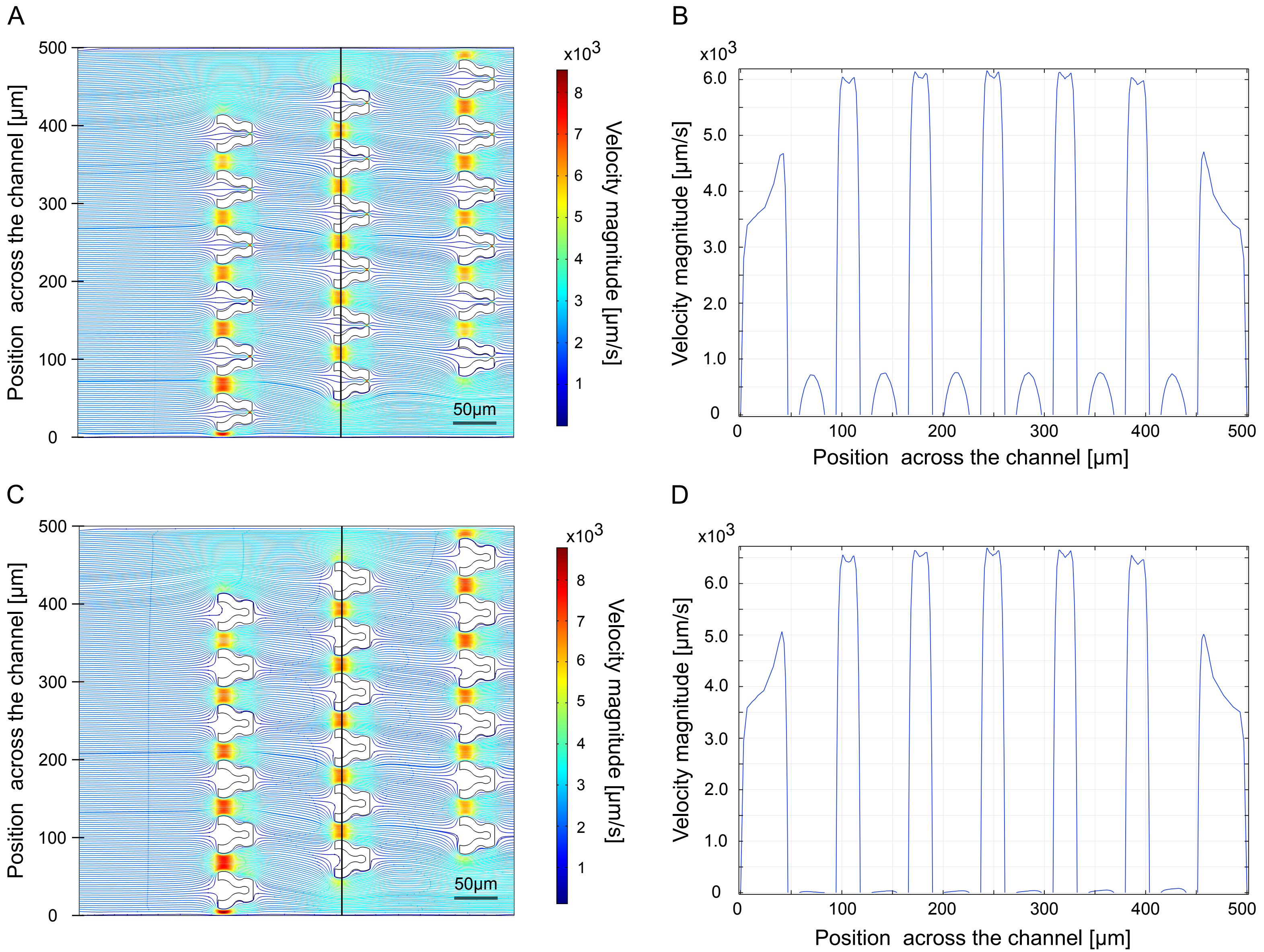

Supplementary Figure S1. Calculated flow field in the “Tulip” microfluidic device. The density of the streamlines and the color code represents the velocity magnitude. A-B) streamlines with empty traps (without trapped cells) and the corresponding velocity magnitude profile across the channel along the line. C-D) streamlines with closed traps (simulating traps occupied by cells) and the corresponding velocity magnitude profile across the channel along the line.
