# Supplementary material for: Microfluidic Platforms Designed for Morphological and Photosynthetic Investigations of Chlamydomonas reinhardtii on a Single-Cell Level
Source: Cells. 2022 Jan 14;11(2):285. doi: 10.3390/cells11020285 (PMC8774182; doi:10.3390/cells11020285)
Supplement: Supplementary file 1 [file cells-11-00285-s001.zip › cells-1507181-supplementary/Supplementary/SupplFigS2_v02.pdf]

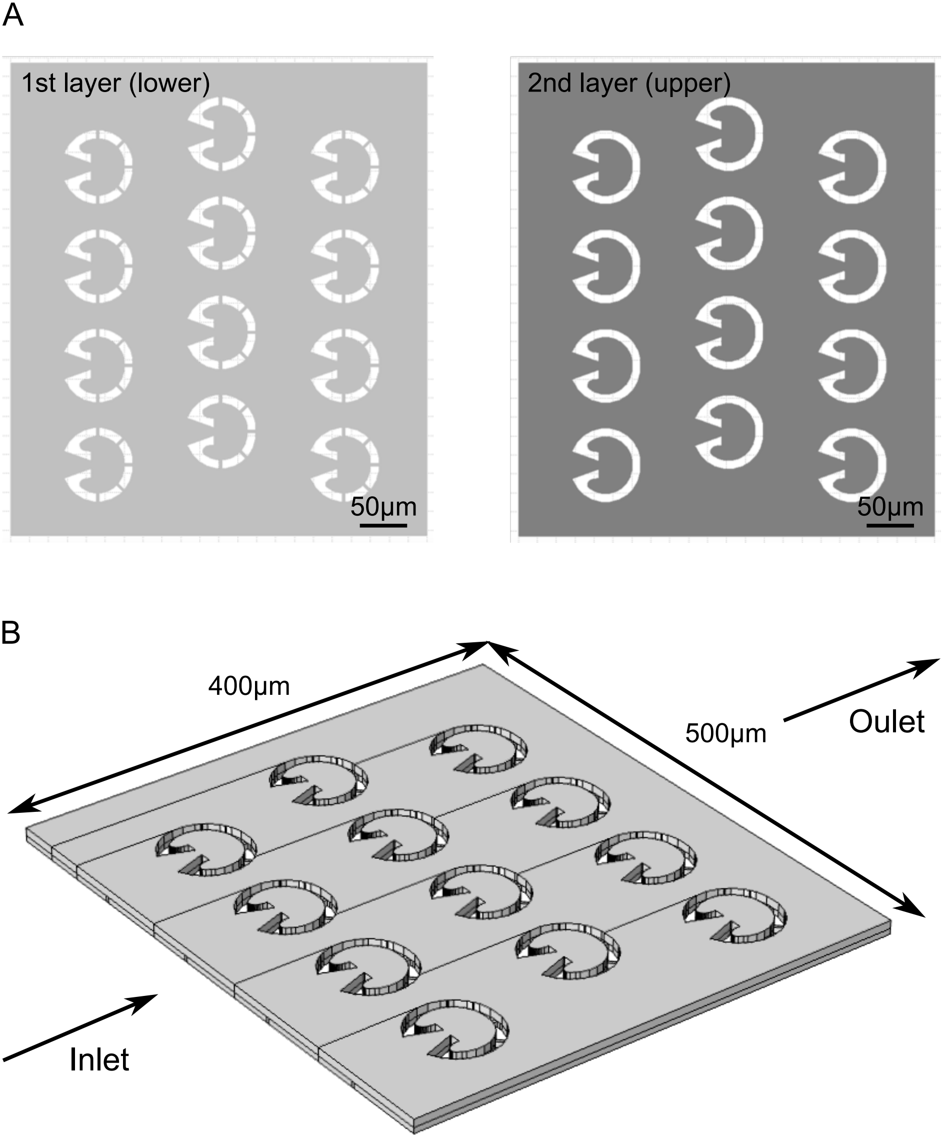

Supplementary Figure S2. Schematic representation of the 3D computational model in case of the two-layered “Pot-shaped” microfluidic device. A) Scheme of the two layers in case of Type VI. Pot-shaped device. B) The applied geometry in the Comsol simulations showing the layered structure and the direction of the fluid flow.
