# Supplementary material for: Microfluidic Platforms Designed for Morphological and Photosynthetic Investigations of Chlamydomonas reinhardtii on a Single-Cell Level
Source: Cells. 2022 Jan 14;11(2):285. doi: 10.3390/cells11020285 (PMC8774182; doi:10.3390/cells11020285)
Supplement: Supplementary file 1 [file cells-11-00285-s001.zip › cells-1507181-supplementary/Supplementary/SupplFigS3_v03.pdf]

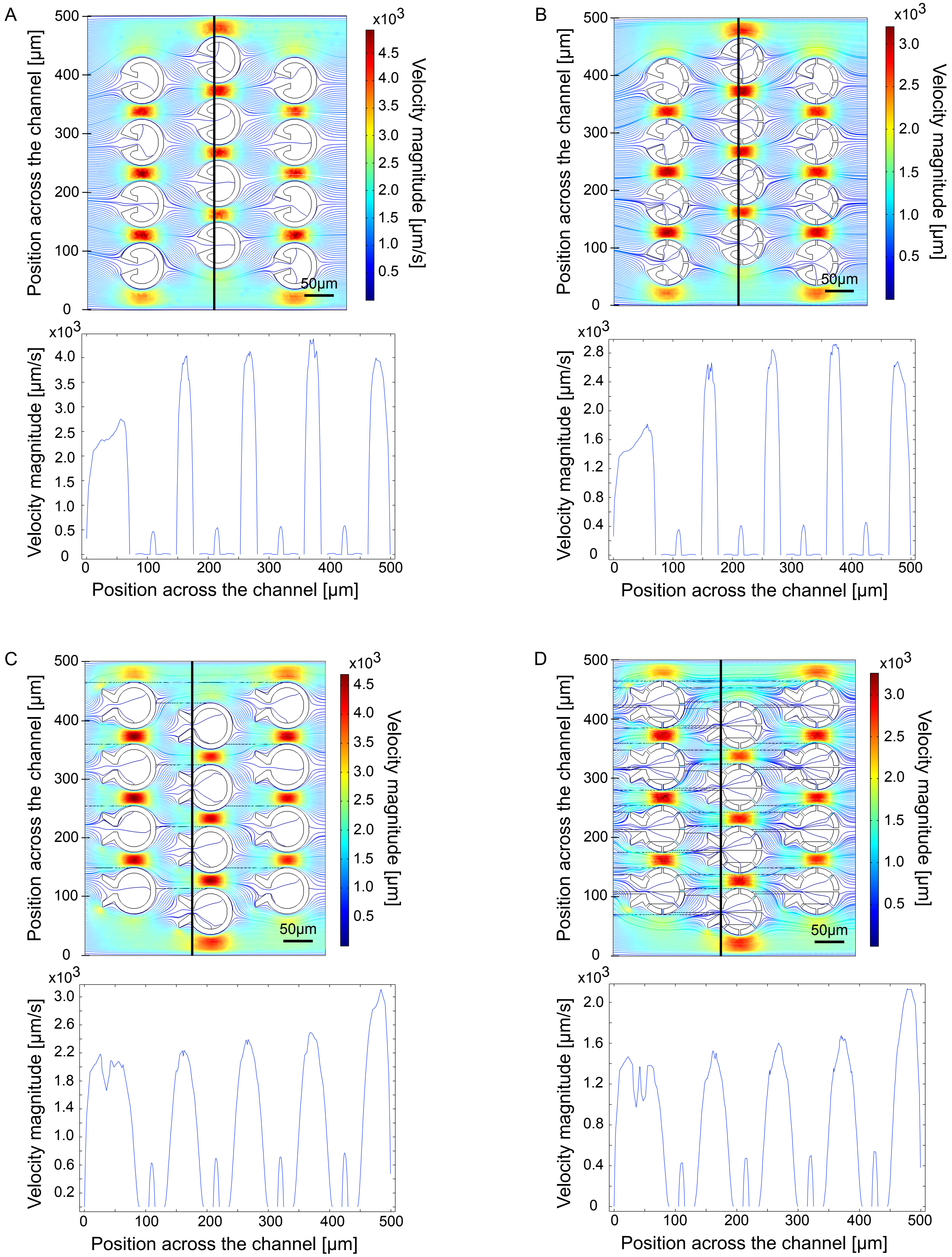

Supplementary Figure S3. Computational modelling of the fluid flow in the “Pot” microfluidic device. The density of the streamlines and the color code represents the velocity magnitude. A-B) flow lines at 7.6 and 1.9 µm depth in case of Type VI traps and the corresponding velocity magnitude profile across the channel along the line;
C-D) flow lines at 7.6 and 1.9 µm depth in case of Type II traps and the corresponding velocity magnitude profile across the channel along the line;
